# Therapeutic Potential of Cholera Toxin B Subunit for the Treatment of Inflammatory Diseases of the Mucosa

**DOI:** 10.3390/toxins9120379

**Published:** 2017-11-23

**Authors:** Joshua M. Royal, Nobuyuki Matoba

**Affiliations:** 1 Department of Pharmacology and Toxicology, University of Louisville School of Medicine, Louisville, KY 40202, USA; joshua.royal@louisville.edu; 2 Center for Predictive Medicine, University of Louisville, Louisville, KY 40202, USA; 3 James Graham Brown Cancer Center, University of Louisville, Louisville, KY 40202, USA

**Keywords:** cholera toxin B subunit, mucosal immunity, immunomodulation, anti-inflammatory, retrograde trafficking, GM1 ganglioside

## Abstract

Cholera toxin B subunit (CTB) is a mucosal immunomodulatory protein that induces robust mucosal and systemic antibody responses. This well-known biological activity has been exploited in cholera prevention (as a component of Dukoral^®^ vaccine) and vaccine development for decades. On the other hand, several studies have investigated CTB’s immunotherapeutic potential in the treatment of inflammatory diseases such as Crohn’s disease and asthma. Furthermore, we recently found that a variant of CTB could induce colon epithelial wound healing in mouse colitis models. This review summarizes the possible mechanisms behind CTB’s anti-inflammatory activity and discuss how the protein could impact mucosal inflammatory disease treatment.

## 1. Introduction

*Vibrio cholerae* is a gram-negative bacterium that can colonize the gastrointestinal tract and cause life-threatening watery diarrhea. The principal virulence factor of *V. cholerae* is cholera toxin (CT), which consists of a catalytic A-subunit and a non-toxic homopentameric B-subunit (CTB) [1,2,3]. CTB binds cells through GM1 ganglioside receptors, which then mediates toxin entry into the cell. It has been previously shown that CTB can induce strong biological activities that can enhance or suppress immune effects under normal and various immunopathological conditions without the toxicity associated with the CTA subunit [4]. Consequently, CTB has been widely studied as a mucosal immunomodulatory agent.

In its most well-known immunostimulatory effects, CTB is used in the vaccine Dukoral^®^. Dukoral^®^ is a WHO pre-qualified oral cholera vaccine which contains heat-killed whole cell *V. cholerae* and recombinant CTB (rCTB). Dukoral^®^ stimulates the production of both antibacterial and antitoxin antibodies, including secretory immunoglobulin A (S-IgA) produced locally in the intestines [5]. CTB itself can induce potent mucosal and systemic antibody response upon mucosal administration in humans [6,7,8], which is largely due to the broad distribution of GM1 ganglioside on various cell types such as epithelial cells, macrophages, dendritic cells (DCs), B cells, T cells, and neurons [9,10,11,12]. Furthermore, the presence of GM1 ganglioside on the luminal surface of intestinal epithelial cells and antigen presenting cells (APCs) in the gut seems to be essential for CTB’s strong mucosal immunostimulatory effects associated with MHC class II expression and local antigen enrichment [13]. In addition, CTB stimulates specific immunosuppressive effects against autoimmune disorders, excess inflammation, and allergic reactions [4,14,15,16,17,18]. We have recently shown that oral administration of a variant of CTB mitigates colitis in chemically-induced acute and chronic colitis mouse models [19]. Although the underlying mechanisms are not well understood, recent studies have shed some light on these immunosuppressive effects induced by CTB. Thus, this review will summarize published studies on CTB’s impacts in mucosal inflammatory disease models, as well as the mechanisms associated with its therapeutic effect and the challenges that CTB faces as an immunomodulatory drug.

## 2. Cholera Toxin Structure and Mechanism in Gut Epithelial Cells

To reveal the mechanism of CTB-induced biological activity, we must first understand the molecule. CT is classified as an AB5 toxin family, which includes the toxins of *Shigella dysenteriae* and enterohaemorrhagic *Escherichia coli*. The toxins are usually composed of one A subunit and five B subunits (CTA and CTB, respectively, for CT). CTA consists of an enzymatically active 11-kDa N-terminal chain (CTA1) and a C-terminal chain (CTA2) that connects CTA to the central pore of CTB. CTB has the capacity to translocate the CTA across the plasma membrane, mediated by the binding of GM1 ganglioside, and then escort CTA from the plasma membrane into the endoplasmic reticulum (ER) [20,21]. The following summarizes CT’s retrograde trafficking mechanism.

The five B-subunits form a central cylindrical pore lined by five amphipathic α-helices that help form a highly stable homopentamer. The pentamer contains five GM1 binding sites that lie on the outer edge of each B subunit [1,22]. Due to an avidity effect from the pentavalent binding capacity, CTB has a very strong affinity (K_D_ reported to be 5 pM to 1 nM) to GM1, which is mainly localized in lipid rafts on the plasma membranes of many cell types [9,10,11,12]. Once CT is bound to GM1 (up to five gangliosides at once), it is endocytosed by clathrin-dependent and independent mechanisms and trafficked via retrograde transport from the Golgi to the ER [21]. It is also known that CT can undergo transcytosis across epithelial cells from the apical to the basolateral surface. However, regardless of how the toxin enters the cell, CT travels to the trans-Golgi network via early endosomal vesicles, independent of the late endosome pathway. The C-terminus of CTA2 possesses a KDEL ER-retention signal for retrieval of CT from the cis-Golgi apparatus to the ER. Interestingly, the KDEL sequence is not vital for retrograde transport of CT to the ER. Mutations that alter the KDEL sequence on CT inhibit KDEL-dependent ER retrieval and decreased (albeit not completely) CT’s toxification [23]. Thus, it is thought that CT’s KDEL sequence—although not absolutely essential—improves the ER’s retrieval of the dissociated CT from the Golgi apparatus and prolongs the time of retention within the ER [20,23,24]. Once in the ER, the CTA1-chain is dissociated from CTA2/CTB complex by protein disulfide isomerase (PDI). Subsequently, CTA1 enters the cytosol via the ER-associated degradation pathway and escapes proteasomal degradation [1,20]. On the other hand, the fate (and remaining function, if any) of CTA2/CTB after releasing CTA1 in the ER is not well documented. Meanwhile, CTA1 reaches the inner surface of the plasma membrane and catalyzes the ADP ribosylation of Gαs, thereby continuously activating adenylate cyclase to produce cAMP. Increased intracellular cAMP impairs sodium uptake and increases chloride outflow, leading to water secretion and diarrhea [20,25].

## 3. At the Cellular Level—What Is Known So Far

**Figure 1:**
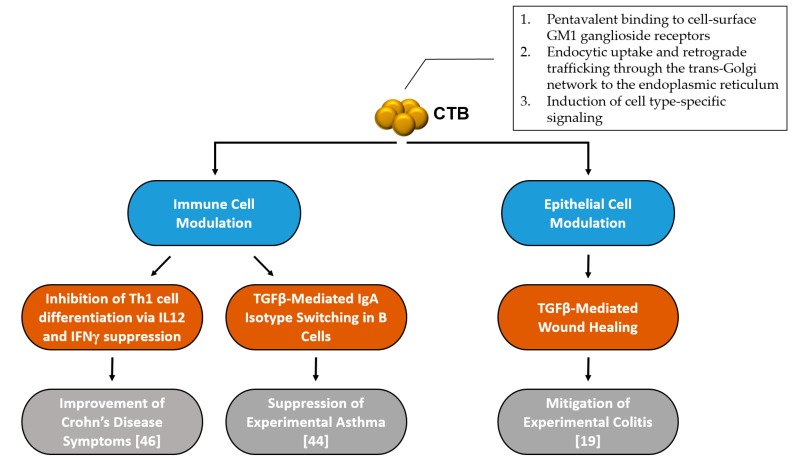
Summary of mechanisms involved in cholera toxin homopentameric B-subunit (CTB)’s inflammatory disease intervention.

Although the virulence mechanism and intracellular trafficking of CT has been well studied, the anti-inflammatory mechanisms of CTB are much less studied and understood. After a comprehensive literature review, it seems that there are at least two separate modes of action induced by CTB to modulate inflammatory responses: one that is based on immune cell regulation, and another that is epithelial cell-mediated (Figure 1).

In 1994, the immune suppressive effects of CTB were first reported by Sun et al. [26]. This report demonstrated that oral administration of mice with CTB conjugated with antigens (sheep red blood cells, horse red blood cells, and human γ-globulin) enhanced oral tolerance to the antigens, presumably through efficient presentation of antigens to immune cells in the gut-associated lymphoid tissue and the generation of regulatory cells. In a Commentary to this article, Weiner suggested that CTB could have enhanced tolerance by serving as a “selective mucosal adjuvant” and that this unique activity could be exploited to treat autoimmunity [27]. Subsequently, this seminal finding led to a new field of studies in which CTB-antigen conjugates were applied to induce tolerogenic reactions to the conjugated antigens in various immunopathological conditions (i.e., encephalomyelitis, autoimmune diabetes, autoimmune arthritis, uveitis) and IgE-mediated allergen hypersensitivity [14,16,17,18,28,29,30,31,32,33,34,35,36,37,38]. Through these studies, it became apparent there are two unique and distinct mechanisms of CTB responsible for the suppression of immunopathological reactions in allergy and autoimmune diseases: (1) to increase antigen uptake and presentation by different APCs through binding to their cell-surface GM1 ganglioside receptors and (2) to induce anti-inflammatory and immunoregulatory activities by directly or indirectly acting on specific immune cells. The latter mechanism points to the possibility that CTB by itself may act as an immunotherapeutic agent; however, only a handful of groups have actually proven that CTB alone—without co-administration or conjugation of antigens—can induce an anti-inflammatory response. Moreover, studies conducted with non-recombinant CTB (nrCTB, prepared by chemically dissociating CTA from CTB) can have significantly skewed experimental results due to trace amounts of CT and CTA [4,39,40]. For example, we have previously shown that picomolar concentrations (<10 ng/mL) of CT significantly inhibited lipopolysaccharide (LPS)-induced TNFα production in RAW264.7 cells, while recombinant (r)CTB failed to induce such an effect at a concentration as high as 10 µg/mL [4]. Thus, the use of rCTB is required to evaluate the effects unique to CTB.

### 3.1. Immune Cell Modulation

With regards to CTB’s immune cell regulation, Kim et al. demonstrated in murine spleen B cells that rCTB dose-dependently increased IgA secretion and inhibited B cell growth [41]. In the presence of IL-2, rCTB significantly increased IgA isotype switching in LPS-activated B cells. These effects were reversed by the addition of an anti-TGFβ or soluble TGFβ1 receptor, which markedly inhibited rCTB-stimulated IgA response. Further analysis in the same report revealed that rCTB stimulated IgA2 B cells, upregulated TGFβ1 mRNA expression, and increased bioactive TGFβ1 levels, which is known to induce IgA isotype switching [41]. Thus, rCTB stimulated a TGFβ-mediated IgA response that was dependent on IL-2 as a cofactor. These findings have contributed to our understandings of how CTB stimulates B cell IgA production, and potentially oral tolerance as well (see below).

It is known that IgA antibodies help maintain mucosal homeostasis and play a role in immune protection [42,43]. Thus, it seems possible that rCTB administration could provide therapeutic effects in mucosal autoimmune disorders via IgA induction. For example, in an experimental mouse model of asthma, nrCTB suppressed the ability of DCs to prime for Th2 responses to inhaled allergen via an IgA-dependent manner [44]. In this study, co-administration of ovalbumin (OVA) and nrCTB suppressed classical features of asthma, including airway eosinophilia, Th2 cytokine synthesis, and bronchial hyperactivity in mice that were pre-sensitized with OVA-stimulated DCs in the lung. Furthermore, nrCTB treatment enhanced DCs’ potential to induce Treg cells in vitro; however, these Treg cells did not provide protection when transferred into the airways of naïve mice that received OVA challenge. In contrast, the transfer of B cells from OVA+CTB-DCs-immunized mice to OVA-sensitized naïve mice significantly reduced eosinophilia and lymphocytosis. It was also found that nrCTB caused a TGFβ-dependent increase in antigen-specific IgA in the airway luminal secretion, and this was attributed to nrCTB’s efficacy against the experimental asthma as the therapeutic effects were abrogated in mice lacking luminal IgA transporter (polymeric Ig receptor), which is necessary for the transport of dimeric IgA across the epithelium into the luminal mucosa [45].

Meanwhile, IgA may not be the sole factor contributing to CTB’s ability to mitigate inflammatory diseases in the mucosa. For example, in the 2,4,6-trinitrobenzene sulfonic acid (TNBS)-induced mouse model of Crohn’s disease, daily oral administration over a four-day period of 100 µg rCTB after the onset of TNBS-colitis immediately resolved weight loss and reduced inflammation [39]. In this case, the timing of mucosal restitution in regard to rCTB administration did not likely result in IgA production. In a similar TNBS-colitis study, rCTB administration reduced IL-12 and IFNγ secretion, inhibited STAT-4 and STAT-1 activation, and downregulated T-bet expression, indicating that rCTB inhibited mucosal Th1 cell signaling [46]. Moreover, these results were confirmed in a small multicenter, open-label, and nonrandomized clinical trial in which 15 patients with active CD received three oral doses of 5 mg rCTB per-week over 2 weeks (six doses total) and were examined 2, 4, 6, and 10 weeks after the start of the study. Of the 12 patients who finished the study per protocol, seven responded to treatment and five were in remission by week six and maintained remission through week 10 as defined by a CD activity index score ≤150 [47]. Of note, side effects seen in 33% of patients administered with CTB were mild (arthralgia, headache, and pruritus), and no safety concerns were raised throughout the trial [47].

Interestingly, rCTB did not reduce disease severity in an oxazolone-induced colitis model performed by the same group [39]. Oxazolone-induced colitis is mediated by IL-4 driven Th2 cells rather than IL-12/IFNγ-driven Th1 cells [39]. Thus, it appears that rCTB administration had a specific effect on specific T cell functions involved in TNBS-colitis [39]. Although the detailed mechanism by which rCTB inhibited Th1 cell was not elucidated, it is possible that the binding of CTB to GM1 ganglioside on immune cells resulted in a signaling cascade of events that led to Th1 inhibition, because non-GM1 binding CTB mutants do not modulate lymphocyte function [48]. In agreement with these findings, rCTB decreased monocyte-derived DC maturation and IL-12 production upon LPS stimulation in vitro [49]. Moreover, rCTB-pretreated, LPS-stimulated DCs induced low proliferating T cells that had enhanced production of IL-10 and reduced production of IFNγ. Rouquete-Jazdanian et al. additionally showed that the binding of rCTB to GM1 ganglioside directly prevented the activation and proliferation of CD4^+^ T cells [50]. This effect was induced by rCTB-mediated sphingomyelinase activation that subsequently increased the production of ceramides, which are known cell cycle arrest inducers [51]. rCTB also inhibited protein kinase Cα, a pro-growth cellular regulator, which was linked to rCTB-induced lipid raft modifications and ceramide-mediated inactivation [52,53].

### 3.2. Epithelial Cell Modulation

Besides serving as a barrier lining the mucosal surface, epithelial cells have multiple functions associated with the maintenance of gut homeostasis and mucosal healing, and crosstalk between epithelial and immune cells is an important component of those complex biological processes [54,55]. Even though CTB first encounters epithelial cells in the gut, the CTB-mediated modulation of epithelial cells and its consequence to the mucosal immune system have largely been ignored in comparison to the protein’s direct impacts on immune cells.

In one small study, CTB was shown to induce a dose-dependent increase of IL-10 mRNA levels in the colon epithelial cell-line T84 [56], hinting that CTB could induce epithelial cell-mediated immune modulation [57]. We have recently characterized CTB’s global impacts on the gut to further our understanding of its unique biological activities. Using a plant-made recombinant CTB (CTBp) [58,59], we have shown that oral administration of the CTB variant significantly altered several immune cell populations in the colon lamina propria [19]. Two-weeks after two oral 30 µg CTBp administrations, Th2 and Treg cells increased in the colon lamina propria. This is not the first report of CTB-induced increase in these cell types [15,36,38,60,61]. For instance, it has been shown that oral administration of a CTB–insulin conjugate in NOD mice induced a shift from Th1 to Th2 profile while generating Treg cells [15]. Additionally, intraperitoneal administration of nrCTB to rats increased Treg cells in the peripheral blood 24–72 h after ischemia [60]. Besides the specific T helper cell subsets, our study has also revealed that innate immune cells—including dendritic cells, natural killer cells and macrophages (both M1 and M2)—populations were increased in the colon lamina propria two weeks after CTBp oral administration [19]. Furthermore, a global gene expression analysis revealed that CTBp had more pronounced impacts on the colon than the small intestine, with significant activation of TGFβ-mediated pathways in the colon mucosa [19]. Given that there is a strong link between epithelial-derived TGFβ and innate immune cells in wound healing [62,63,64], the results provided implications for the potential utility of CTBp to promote colonic mucosal health. Subsequently, we found that CTBp induced TGFβ-mediated wound healing in Caco2 colon epithelial cells. Furthermore, oral administration of CTBp in mice protected against colon mucosal damage in acute colitis induced by dextran sodium sulfate (DSS). Two oral doses of as low as 1 µg of CTBp mitigated clinical signs of disease (body weight loss, decreased histopathological scores, and blunted escalation of inflammatory cytokine levels) and upregulated wound healing-related genes [19]. Interestingly, CTBp administration prevented fibrosis associated with acute colitis in mice; hence, the protein did not appear to overstimulate TGFβ signaling. In fact, TGFβ gene expression levels were high during the early inflammatory phase and became lower in the recovery phase of the acute colitis model in CTBp-treated mice.

In contrast to TNBS-induced colitis, the DSS-colitis model closely approximates human ulcerative colitis (UC) [65,66,67,68]. Thus, the results point to the possibility that CTBp could be used to facilitate mucosal healing in the management of UC. Since the main driver of intestinal inflammation in the DSS model is the damage to the epithelial barrier lining the colon that allows intestinal microbiota into submucosal compartments [69], and since therapeutic effects were observed immediately upon CTBp administration, we concluded that CTBp’s protective efficacy in the DSS colitis models were attained by the induction of TGFβ-mediated colonic epithelial wound healing. Given that UC poses an increased risk of developing colitis-associated colorectal cancer (CAC) [70,71], CTBp’s effects were also examined in the azoxymethane (AOM)/DSS mouse model of CAC. Biweekly oral administration of CTBp over 9 weeks significantly reduced inflammation and tumorigenesis in this model, again highlighting its therapeutic potential in UC treatment [19].

It is of importance to point out that many of the effects observed in the aforementioned studies using CTBp may be unique to the plant-made variant, as it has a mutation at amino acid position 4 and an ER retention signal sequence at the C-terminus (N4S-CTB-SEKDEL; [58]). The ER-retention sequence was added to CTBp to improve production in planta, while Asn4→Ser mutation was introduced to avoid *N*-glycosylation [58,59]. The addition of the KDEL sequence to N4S-CTB significantly reduced ER stress that otherwise caused poor production yield. It is thought that the KDEL sequence helped prolong CTBp’s residence time in the ER to allow for proper folding and assembly.

The protein ER retention mechanism involving the KDEL receptor is highly conserved among eukaryotic organisms [72]. Thus, there is a possibility that the artificial KDEL sequence of CTBp may prolong the protein’s residence in the epithelial cells upon binding to cell-surface GM1 ganglioside and retrograde transport into the ER, as has been demonstrated for CT [21,23]. Subsequently, this may induce a level of altered cell signaling. For example, interaction between CTBp’s C-terminal KDEL sequence and KDEL receptors may have an impact on ER homeostasis [73,74]. The binding of proteins to the KDEL receptor and the induction of mild UPR have been linked to TGFβ activation, wound healing, colon epithelial cell prosurvival signaling, and protection from DSS-induced colitis [73,75,76,77]. Of note, CT is a known inducer of the UPR in epithelial cells due to the KDEL sequence on CTA [23,78,79], while CTB has no effect on the UPR or ER signaling [78].

Regardless of whether the ER retention signal had a significant contribution to the mucosal healing activity in the mouse colitis models, the study has provided evidence that CTB can exhibit a therapeutic effect against colitis in an epithelia-dependent manner, warranting further investigation of CTB’s impacts on epithelial cells.

## 4. Conclusions—Challenges for the Use of CTB as an Immunomodulatory Drug

Although CTB has been administered in humans in the form of oral cholera vaccines over the past two decades, its development as an immunomodulatory drug will need to address unique issues associated with therapeutic use besides additional testing of safety and efficacy in specific disease indications. One of the principal questions is whether CTB’s strong mucosal immunogenicity that induces a robust IgG and IgA immune response [4,58] is beneficial or dispensable to its anti-inflammatory/immunosuppressive effects. From a conventional biopharmaceuticals development standpoint, anti-drug antibodies constitute a theoretical risk because they may affect drug efficacy and pharmacokinetics, and potentially cause immunotoxicity [80,81]. However, induction of an antibody response—particularly that of IgA isotype—may play an important role in mitigating mucosal inflammation, as illustrated in the asthma study described in Section 3.1 [44]. The CD clinical trial showed an efficacy up to 10 weeks after repeated CTB administrations over 2 weeks [47]. Although not reported, the treatment regimen must have elicited high levels of anti-CTB antibodies in the gut and blood circulation. Thus, further investigation is necessary to address long-term efficacy following repeated CTB dosing. TGFβ seems to be a major denominator of CTB-induced immunomodulatory activities. TGFβ is a pleiotropic cytokine playing critical roles in cell differentiation and proliferation, as well as dynamic biological processes in wound healing and immune responses [82,83,84]. The cytokine is also involved in various pathological conditions. For example, elevated TGFβ levels have been correlated to the development of fibrosis following injury to the skin [85]. TGFβ mediates epithelial-to-mesenchymal transition (EMT) [86], and reduction of TGFβ1 levels in a mouse model of pulmonary fibrosis blunted fibrosis [87]. TGFβ signaling also has important implications in cancer. Although the cytokine functions as a suppressor of tumorigenesis at an early stage of tumor development, its expression is correlated with tumor progression and poor prognosis at late stages [84,88]. Collectively, the double-edged sword nature of TGFβ points to the importance of careful investigation of possible consequences upon long-term CTB dosing for the treatment of chronic inflammatory diseases. As mentioned in Section 3.2, CTBp treatment significantly mitigated gut inflammation and reduced tumor development in a model of CAC [19], providing a basis for further investigations of long-term therapeutic use of CTB for the treatment of IBD.

Of considerable interest may be population-based studies investigating potential association between the Dukoral^®^ vaccine and gastrointestinal disorders involving mucosal inflammation. In a very recent study of patients who were diagnosed with colorectal cancer from July 2005 through December 2012 in Sweden, it was revealed that those who had previously received Dukoral^®^ had a significantly reduced risk of death from colorectal cancer (CRC) [89]. Although the underlying mechanism is not clear at this point, the authors speculated that CTB might be associated with a risk reduction of CRC [89]. This observation warrants a comprehensive investigation on this subject.

In conclusion, even though CTB has been studied since the early 1970s [3], its immunomodulatory mechanisms appear to involve complex interplay between epithelial and immune cells that requires a systematic approach for comprehensive understanding. The studies highlighted herein strongly suggest CTB’s potential as an effective mucosal anti-inflammatory agent with the potential to replace or supplement currently available therapies for the treatment of inflammatory disorders of the mucosa, such as anti-TNFα biologics used in IBD patients who are refractory to conventional medications. As anti-TNFα agents are administered systemically, these agents have limited efficacy for the induction of mucosal healing [90,91] and/or pose severe adverse reactions [92,93,94]. In contrast, CTB has few, if any, adverse effects (according to the CD clinical trial [47]), can directly heal lesions/ulcers, and blunt inflammation upon topical administration. Therefore, to aid in developing CTB-based therapeutic strategies against various mucosal immunopathological conditions, further research that delineates how CTB can modulate epithelial cell signaling and T cell functions simultaneously is warranted.
